# Tracking research trends and hotspots in sperm DNA fragmentation testing for the evaluation of male infertility: a scientometric analysis

**DOI:** 10.1186/s12958-019-0550-3

**Published:** 2019-12-26

**Authors:** Saradha Baskaran, Ashok Agarwal, Manesh Kumar Panner Selvam, Renata Finelli, Kathy Amy Robert, Concetta Iovine, Peter Natesan Pushparaj, Luna Samanta, Avi Harlev, Ralf Henkel

**Affiliations:** 1 American Center for Reproductive Medicine, Cleveland Clinic, Mail Code X-11, 10681 Carnegie Avenue, Cleveland, OH 44195 USA; 2 Center of Excellence in Genomic Medicine Research, King Abdulaziz University, Jeddah, Kingdom of Saudi Arabia; 3 Department of Medical Laboratory Technology, Faculty of Applied Medical Sciences, King Abdulaziz University, Jeddah, 21589 Kingdom of Saudi Arabia; 4 Redox Biology Laboratory, Center of Excellence in Environment and Public Health, Ravenshaw University, -753003, Cuttack, India; 5 Department of Obstetrics and Gynecology, Soroka University Medical Center, Ben-Gurion University of the Negev, Beer-Sheva, Israel; 6 Department of Medical Bioscience, University of the Western Cape, Bellville, South Africa

**Keywords:** Scientometric analysis, Diagnosis, DNA fragmentation, Infertility, male, Prognosis, Publications

## Abstract

**Background:**

This article describes the research trends in sperm DNA fragmentation (SDF) over the past 20 years (1999–2018) using a scientometric approach.

**Methods:**

A stepwise approach was adopted to retrieve scientometric data (articles per year, authors, affiliations, journals, countries) from Scopus and analyze the publication pattern of SDF with reference to key areas of research in the field of Andrology.

**Results:**

A total of 2121 articles were retrieved related to SDF. Our data revealed an increasing research trend in SDF (*n* = 33 to *n* = 173) over the past 20 years (R^2^ = 0.894). Most productive country in publications was the USA (*n* = 450), while Agarwal A. (*n* = 129) being the most productive author. Most of the articles in SDF were primarily focused on lifestyle (*n* = 157), asthenozoospermia (*n* = 135) and varicocele (130). Mechanistic studies on SDF were published twice as much as prognostic/diagnostic studies, with significant emphasis on oxidative stress. Terminal deoxynucleotidyl transferase dUTP nick end labeling (TUNEL) was the most widely used technique to evaluate SDF. Publications on SDF related to assisted reproductive techniques also showed a linear increasing trend (R^2^ = 0.933).

**Conclusions:**

Our analysis revealed an increasing trend in SDF publications predominantly investigating lifestyle, asthenozoospermia and varicocele conditions with TUNEL being the most widely used technique. A substantial increase in research is warranted to establish SDF as prognostic/diagnostic parameter to evaluate clinical scenarios and ART outcomes.

## Background

Global prevalence of infertility in couples of reproductive age is about 15% and 50–70% of these cases are associated with male factor [1, 2]. Semen analysis is the cornerstone for male infertility assessment; however, it fails to predict the reproductive outcome [3]. The integrity of paternal genome is vital for fertilization and formation of healthy offspring. After the fertilization, sperm DNA starts to transcribe actively at the 4-cell stage, contributing to 50% of the embryonic genome [4]. Therefore, sperm DNA integrity in ejaculated sperm is an imperative factor for successful fertilization, embryo development, implantation and pregnancy. In 1980, Evenson et al. introduced sperm chromatin structure assay (SCSA) for the evaluation of sperm DNA damage [5]. Since then, several different methods have been developed to assess sperm DNA fragmentation (SDF) such as terminal deoxynucleotidyl transferase dUTP nick end labeling (TUNEL), Comet assay and sperm chromatin dispersion test (SCD) [6].

SDF can originate in both testicles and during sperm transit in the genital tract [7]. Increased levels of seminal apoptotic M540 bodies suggest disruption of spermatogenesis and testicular abnormalities [8]. Two populations of sperm with different extent of DNA fragmentation (PI^brighter^ and PI^dimmer^) have been identified to be important with respect to clinical investigations [9, 10]. SDF in PI^dimmer^ population has been correlated with semen quality, while DNA fragmentation in PI^brighter^ sperm is associated with clinical and ultrasound characteristics of male genital tract [7]. High levels of DNA damage have been reported in various conditions such as varicocele, unexplained male infertility (UMI), and idiopathic male infertility [11–15]. Several studies and meta-analysis have reported the detrimental effects of sperm DNA damage on the reproductive outcomes via artificial reproductive techniques (ART) [16–18]. Moreover, a significant association between sperm DNA damage and pregnancy failure has been reported [19, 20]. Normozoospermic male partners of couples experiencing unexplained recurrent pregnancy miscarriages had high percentage of sperm DNA damage [21]. The limitations of standard semen analysis in assessing the fertilizing potential of male gametes led to an increased attention on the significance of SDF in providing a better molecular understanding of male infertility. Though routine application of SDF analysis is not recommended, professional societies such as American Society for Reproductive Medicine (ASRM), American Urological Association (AUA) and the European Association of Urology (EAU) have acknowledged the importance of SDF testing in the evaluation of male infertility [22, 23]. In the current scenario, application of SDF testing is one of the prime topics of discussion and controversy in the field of Andrology, which intrigued us to analyze the research trends in SDF over the past 20 years.

Scientometrics is a quantitative analysis of scientific literature aimed at measuring and analyzing several aspects of published documents such as institutional and author productivity, networking, and impact analysis of journals, authors and regions [24, 25]. It is a useful tool to monitor the publication trend, which reflects the growth pattern in a particular field of interest [26, 27]. Our earlier publication has provided an in-depth report on male infertility research by analyzing the scientometric data (1998–2017) retrieved from Scopus using a Funnel Model [27]. The analysis shed light on various key elements, including the growth of OMICS and ART in male infertility research [27]. To date, there are no publications on research trends in SDF. Therefore, the main objectives of our study was to conduct a comprehensive, stepwise analysis of the literature in order to delineate publication trends in (a) SDF, (b) SDF-associated male infertility studies, (c) clinical scenarios and risk factors associated with SDF, (d) mechanistic and prognostic/diagnostic studies of SDF, (e) SDF using specific evaluation techniques and (f) SDF-based ART research. The stepwise analysis allows the study of publication pattern on SDF with reference to various important and pertinent areas of research in the field of Andrology.

## Materials and methods

### Data source

In this study, we used Scopus, the most comprehensive bibliographic database, to retrieve data (https://www-elsevier-com.ccmain.ohionet.org/solutions/scopus/how-scopus-works/content). Scopus is a multidisciplinary database having extensive journal coverage, largest number of abstracts and about 1.4 billion cited references extending back to 1970. About 71 million core records are present in Scopus, which is updated biweekly. Over 3 million new items are added to the database yearly after the independent Scopus Content Selection and Advisory Board (CSAB) review. Scopus can analyze the search results and provide metrics on the number of documents by year, author, affiliation, journal, country or territory, type of document, subject area and number of citations, which are essential for scientometric analysis. Furthermore, Scopus provides author rank in a particular area of research, as well as the Hirsch-index (h-index) of the authors [28, 29].

### Data retrieval strategy

**Fig. 1:**
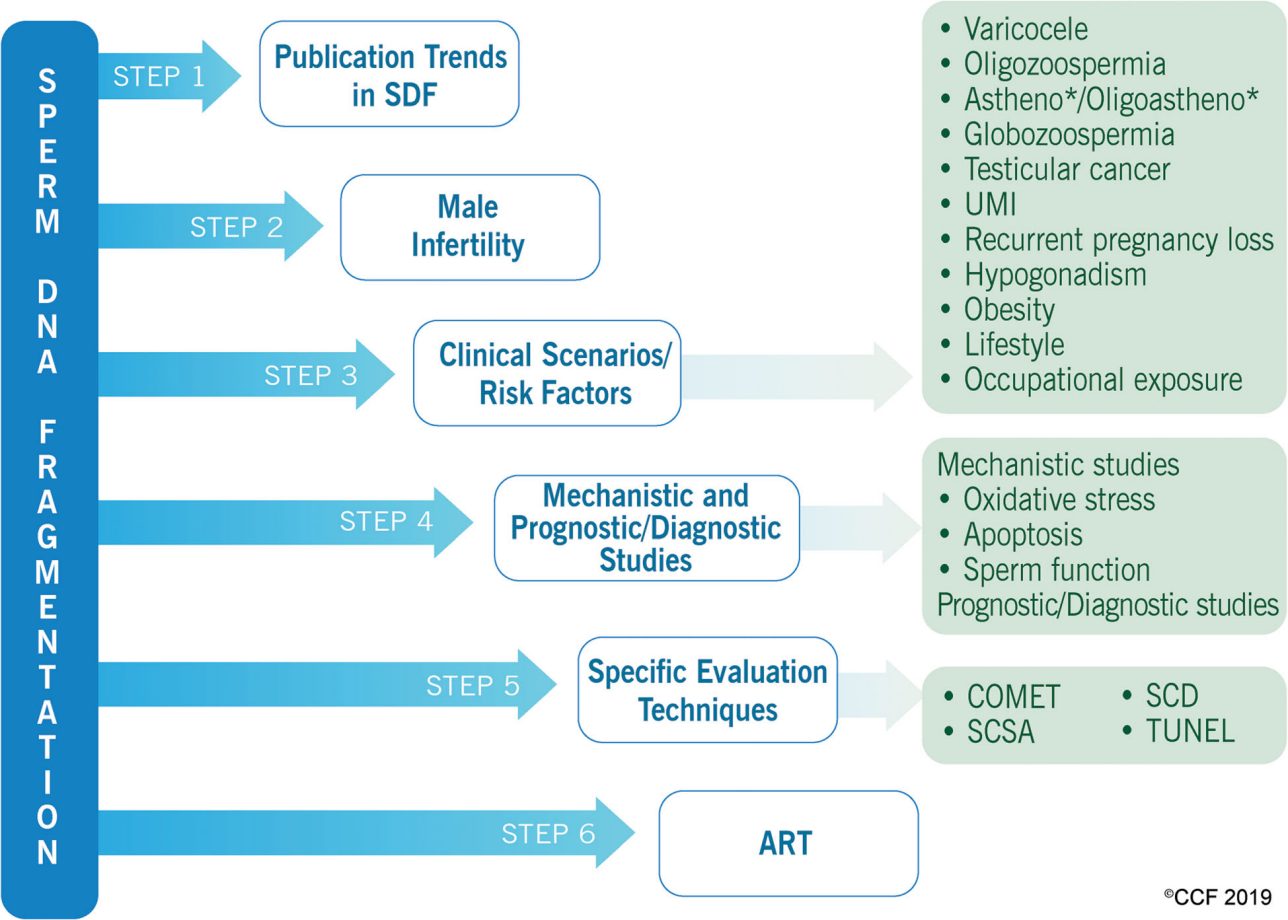
Flow diagram depicting the stepwise analysis of publication trends in SDF. Subcategory “Astheno*/Oligoastheno*” includes articles on asthenozoospermia, asthenoteratozoospermia, oligoasthenozoospermia, and oligoasthenoteratozoospermia. UMI: unexplained male infertility, SCSA: sperm chromatin structure assay, SCD: sperm chromatin dispersion test, TUNEL: terminal deoxynucleotidyl transferase dUTP nick end labeling, ART: artificial reproductive techniques

The literature search in Scopus was conducted on July 25, 2019. Since the 2019 data do not represent the entire year, the search was limited to scientific articles on human subjects published from 1999 to 2018. We used the asterisk ‘*’ after the word to include all variants of the term as well as multiple Boolean operators such as ‘AND’, ‘OR’, ‘NOT’ and ‘AND NOT’ to annul false-positive results. Also, functions such as ‘TITLE-ABSTRACT’ and ‘TITLE-ABSTRACT-KEYWORDS’ were used to retrieve a maximum number of relevant articles. The search was performed in six sequential steps as illustrated in Fig. 1. The keywords for each step (listed in Additional file 3: Table S1) were validated by three independent researchers upon reviewing the title and abstract of all retrieved articles for relevance. The non-human studies as well as articles non-specific to the topic for each step were enlisted as irrelevant articles. The combined percentage of irrelevant articles from three independent researchers was less than 5% for most steps, which were excluded from the analysis.

In this analysis, step 1 included all the scientometric data available on SDF from 1999 to 2018. For the subsequent steps, scientometric data were retrieved using the search option used in step 1 along with additional keywords corresponding to the respective steps (Additional file 3: Table S1). In step 2, additional keywords (Infertil* OR Subfertil* OR Sterility) were used to exclusively narrow down the articles related to ‘SDF and male infertility’. Step 3 was divided into 11 sub-categories, which included different clinical scenarios such as varicocele, oligozoospermia, asthenozoospermia / asthenoteratozoospermia / oligoasthenozoospermia / oligoasthenoteratozoospermia (here after, it is collectively referred as astheno*/oligoastheno*), globozoospermia, testicular cancer, UMI, recurrent pregnancy loss, hypogonadism, and risk factors (obesity, lifestyle and occupational exposure) associated with SDF and male infertility [27]. In step 4, SDF articles related to mechanistic and prognostic/diagnostic studies were retrieved using specific keywords along with the search option used in step 1. Studies that are designed to understand a biological process, pathophysiology, or the mechanism of action of intervention are described as mechanistic by National Institute of Health (NIH) (https://www.niaid.nih.gov/grants-contracts/determine-whether-nih-considers-your-mechanistic-study-clinical-trial). Hence, the articles involving oxidative stress, apoptosis and sperm function were retrieved under mechanistic studies. For step 5, articles on the four major techniques used to assess SDF, namely TUNEL, SCSA, Comet and SCD, were obtained [6]. In the last step, scientometric data on SDF and ART were retrieved.

### Scientometric analysis

The scientometric data for the number of documents based on the year of publication, subject area, journal, country or territory, author, affiliation and document type were retrieved from the Scopus database. This data has a default threshold value of 15 for each operating function. These comma-separated value (CSV) files were converted and saved as Microsoft Excel files for descriptive statistical analysis. The geographic mapping based on the scientometric analysis of research trends in SDF across the globe was done using Tableau Desktop (Tableau, Seattle, USA).

Linear regression analysis was used to investigate the publication trend in the SDF research from 1999 to 2018. All the statistical analysis was carried out using Microsoft Excel (2013).

### Network and heat map analysis

Network map on international collaborations in SDF research were analyzed using VOS viewer (downloaded from http://vosviewer.com) software [30]. The relatedness of the countries was determined based on the number of co-authored documents while the number of documents published by each country defined the size of the nodes. The same software was used to generate the heat maps illustrating the top scientists and the journals in the area of SDF and male infertility (step 2).

## Results

### Step 1 - publication trends in SDF research

**Fig. 2:**
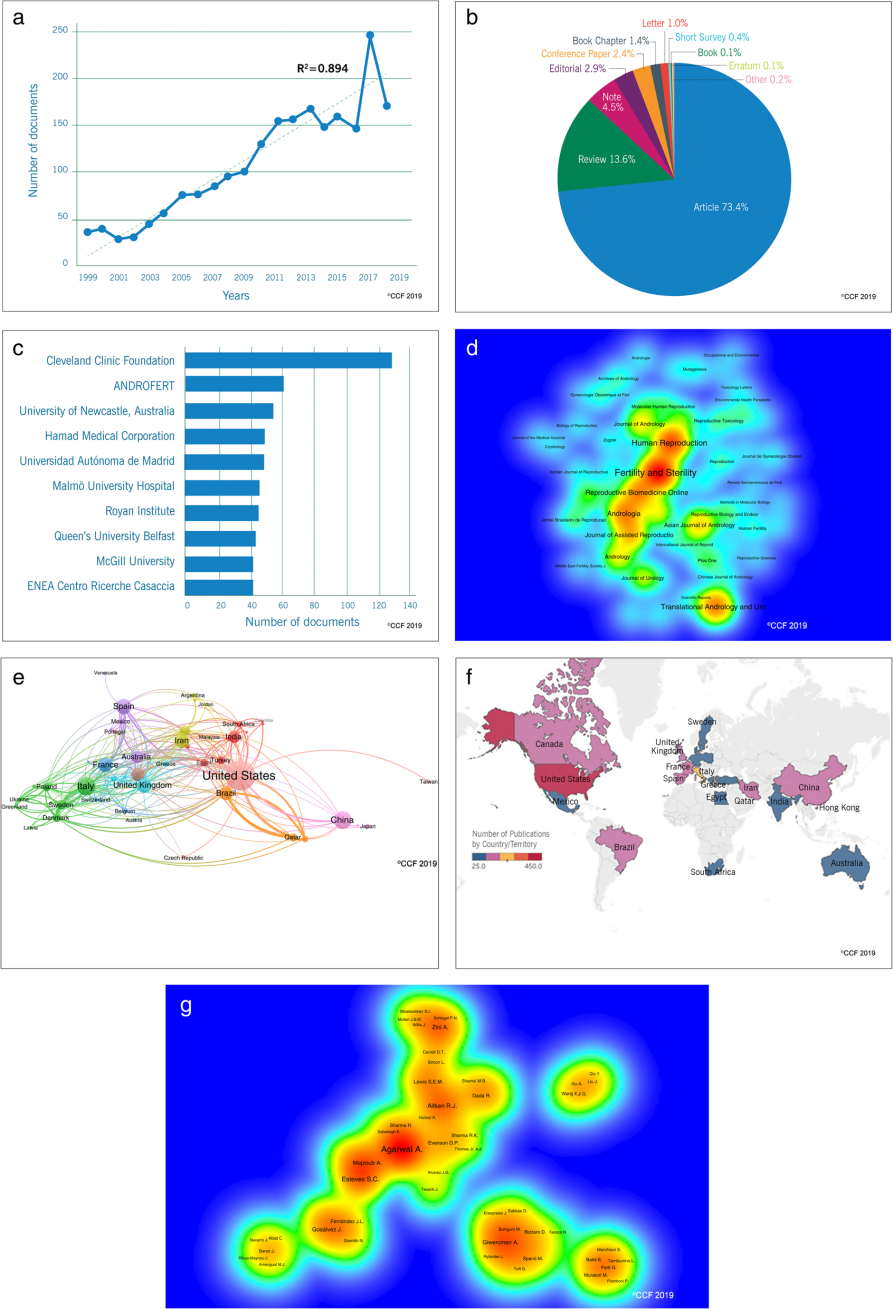
**a** Publication trends in SDF-based research over the past 20 years. **b** Analysis of the research trends based on types of publication. **c** Top 10 institutions based on the publications in SDF (1999–2018). **d** Contour/Heat map showing the publications in SDF (1999–2018). **e** International collaborations in SDF research. **f** Analysis of the research trends based on the origin of publications across the globe. **g** Contour/Heat map showing the top scientist working in the area of SDF. Heat map 2d and 2g shows gradient heat pattern (red, orange, yellow, green and blue) based on the number of publications from journal and author, respectively. The font size of the journal and author names are presented in an ascending manner with respect to an increasing number of publications. Only the names of journals and authors having higher number of publications are visible

In the past 20 years, a total of 2121 articles related to SDF were published. We noted a linear increasing trend (R^2^ = 0.894) in publication from 1999 (*n* = 33) to 2018 (*n* = 173) (Fig. 2a). The majority of the publications were original articles and reviews (Fig. 2b). Furthermore, we identified Cleveland Clinic Foundation in USA, Androfert in Brazil and University of Newcastle in Australia as the top 3 institutions conducting extensive research on SDF (Fig. 2c). Analysis of Scopus results using VOS viewer software revealed Fertility and Sterility (*n* = 209, 9.9%), Human Reproduction (*n* = 146, 6.9%) and Translational Andrology and Urology (*n* = 110, 5.2%) as top journals publishing articles on SDF (Fig. 2d). The USA was identified as the most active country in research collaboration and productive in terms of publications (*n* = 450, 21.2%), followed by Italy (*n* = 195, 9.2%) and China (*n* = 184, 8.7%) (Fig. 2e and f). Analysis of authors contribution revealed Agarwal, A. (*n* = 129) as the most productive scientist in the field of SDF research, followed by Esteves, S.C. (*n* = 66) and Aitken, R.J. (*n* = 57) (Fig. 2g).

### Step 2 - publication trends in SDF-associated male infertility studies

**Fig. 3:**
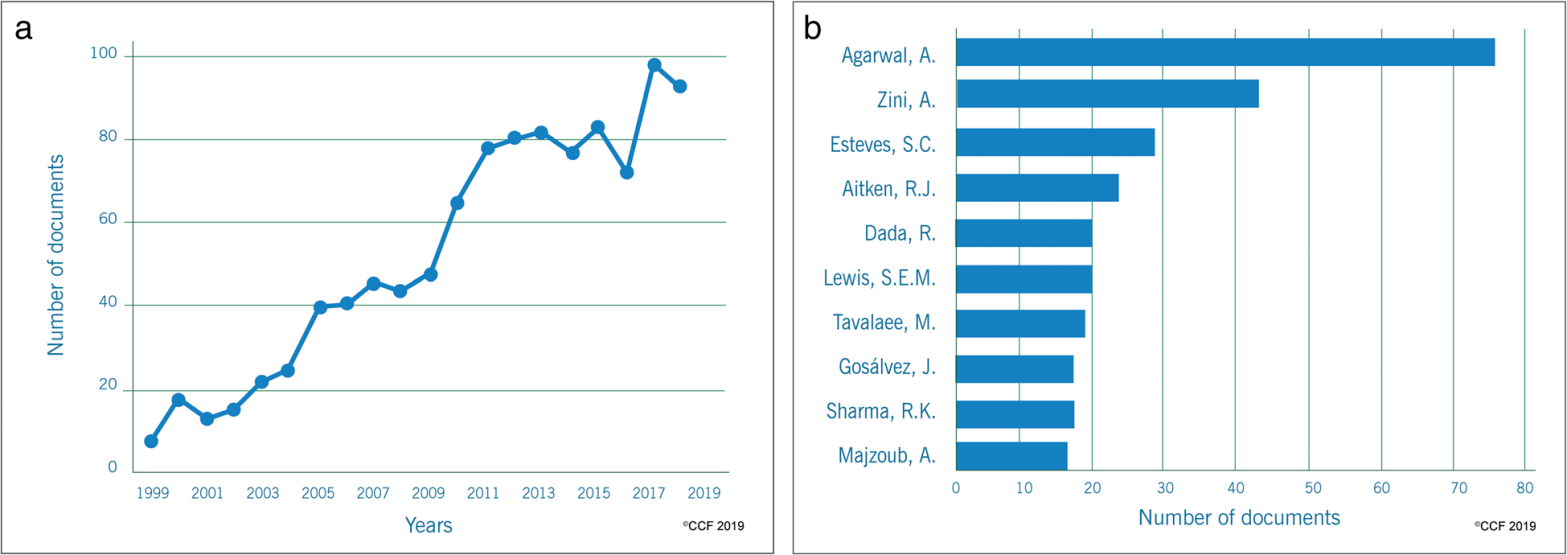
**a** Research trends on SDF-associated male infertility studies. **b** Top scientist investigating the role of SDF in male infertility

Our scientometric analysis retrieved 1038 out of 2121 (49%) articles reporting the association between SDF and male infertility. Research publications on SDF in male infertility have remarkably increased from 1999 (*n* = 7) to 2018 (*n* = 93), particularly in the past 10 years (Fig. 3a). Analysis of the authors contribution revealed the top 3 researchers as Agarwal, A., Zini, A., and Esteves, S.C. (Fig. 3b). In terms of the document type, 75.4% were original articles. Analogous to the step 1, the top journals were identified to be Fertility and Sterility (*n* = 106) and Human Reproduction (*n* = 74) publishing the highest number articles on SDF and male infertility research.

### Step 3 - publication trends in clinical scenarios/risk factors associated with SDF

**Fig. 4:**
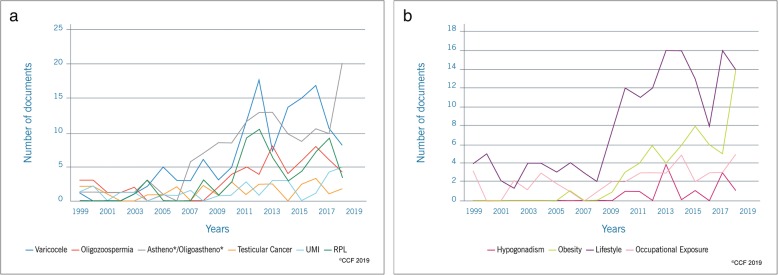
**a** Publication trends on SDF based research on varicocele, oligozoospermia, Astheno*/Oligoastheno*, testicular cancer, unexplained male infertility (UMI), recurrent pregnancy loss (RPL). Astheno*/Oligoastheno* includes articles on asthenozoospermia, asthenoteratozoospermia, oligoasthenozoospermia, and oligoasthenoteratozoospermia. **b** Publication trends on SDF-based research on hypogonadism, obesity, lifestyle and occupational exposure

Publications on SDF were investigated considering different clinical scenarios (astheno*/oligoastheno*, oligozoospermia, UMI, globozoospermia, hypogonadism, recurrent pregnancy loss - RPL, testicular cancer, varicocele) and risk factors (lifestyle, obesity, occupational exposure) (Additional file 1: FigureS1, Fig. 4a and b). According to our results, lifestyle (*n* = 157), astheno*/oligoastheno* (*n* = 135) with asthenozoospermia (*n* = 71) (Additional file 2: Figure S2), and varicocele (*n* = 130) were the top 3 areas where SDF was extensively investigated while testicular cancer (*n* = 28), globozoospermia (*n* = 18) and hypogonadism (*n* = 11) had received less attention (Additional file 1: Figure S1).

### Step 4 - publication trends of mechanistic and prognostic/ diagnostic studies on SDF

**Fig. 5:**
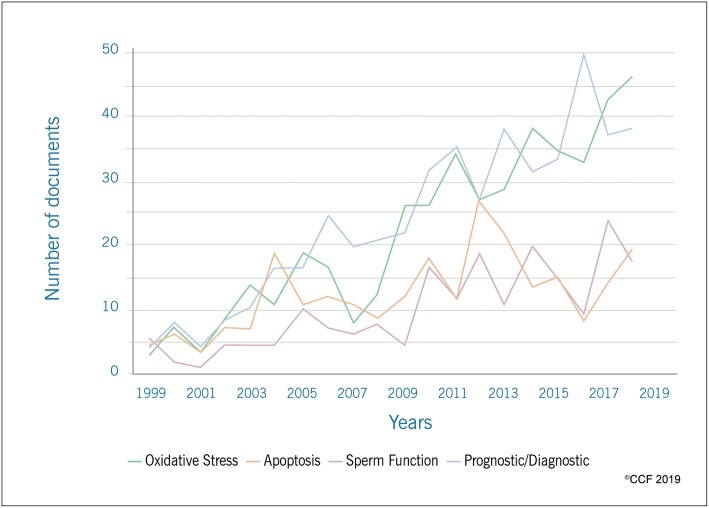
Publication trends in mechanistic studies and prognostic/diagnostic studies on SDF

Articles on SDF were further sub-categorized into mechanistic and prognostic/diagnostic studies (Fig. 5). Literature on oxidative stress, apoptosis and sperm function were considered as mechanistic studies. For mechanistic studies, original articles accounted for 72.3% and reviews for 20.4% with maximum publications from USA. The most productive authors are listed in Table 1. Of the mechanistic studies, a great emphasis was on oxidative stress, accounting for 50.81% of total publications.

**Table 1:** Top scientists, institutions and countries involved in mechanistic and prognostic/diagnostic studies on SDF

	Prognostic/Diagnostic (*n* = 439)	Oxidative stress (*n* = 438)	Apoptosis (*n* = 234)	Sperm function (*n* = 190)
Top Scientists	Agarwal, A. (*n* = 25)	Agarwal, A. (*n* = 45)	Agarwal, A. (*n* = 16)	Aitken, R.J. (*n* = 17)
	Giwercman, A. (*n* = 14)	Aitken, R.J. (*n* = 44)	Aitken, R.J., Calogero, A.E., & Vicari, E. (*n* = 13)	Agarwal, A. (*n* = 13)
	Evenson, D.P. & Lewis (*n* = 12)	Dada, R. (*n* = 23)	La Vignera, S. (*n* = 9)	Calogero, A.E. & Zini, A. (*n* = 6)
Top Countries	United States (*n* = 97)	United States (*n* = 85)	United States (*n* = 46)	United States (*n* = 30)
	Italy & Spain (*n* = 40)	Australia (*n* = 51)	Italy (*n* = 35)	Italy & Australia (*n* = 21)
	France (*n* = 33)	India (*n* = 45)	Iran (*n* = 24)	Iran(n = 20)
Top Institutions	Cleveland Clinic Foundation (*n* = 25)	University of Newcastle, Australia (*n* = 42)	Cleveland Clinic Foundation & Università degli studi di Catania (*n* = 15)	University of Newcastle, Australia (*n* = 16)
	South Dakota State University & Queen’s University Belfast (*n* = 15)	Cleveland Clinic Foundation (*n* = 41)	University of Newcastle, Australia (*n* = 13)	Cleveland Clinic Foundation (*n* = 12)
	Malmö University Hospital (*n* = 14)	All India Institute of Medical Sciences, New Delhi (*n* = 26)	Shahid Sadoughi University of Medical Sciences (*n* = 8)	McGill University & Università degli studi di Catania (*n* = 7)

Publications on diagnostic/prognostic value of SDF accounted for 33.74% of the total number of publications, showing a growing trend with a maximum number of articles in 2016 (*n* = 46) (Fig. 5). Human Reproduction (*n* = 49), Fertility and Sterility (*n* = 40) and Reproductive Biomedicine Online (*n* = 23) together published 25.51% of the articles. The top scientists, institutions and countries involved in the diagnostic/prognostic studies on SDF are listed in Table 1.

### Step 5 - publication trends in SDF using specific evaluation techniques

**Fig. 6:**
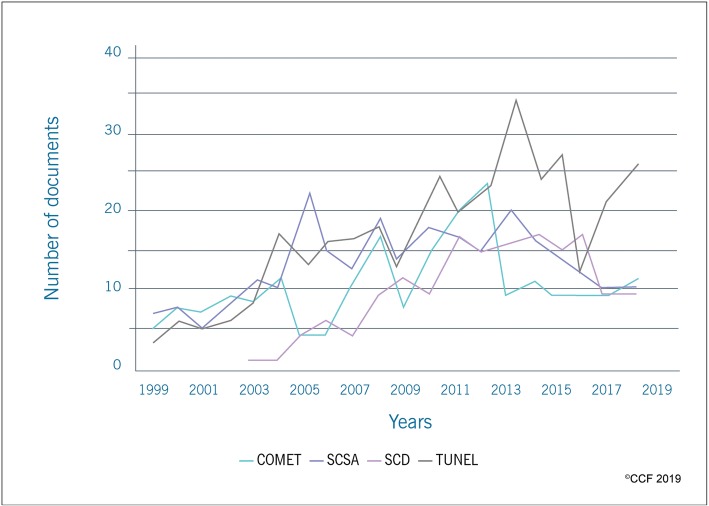
Publication trend in SDF using TUNEL/SCSA/Comet/SCD between1999–2018

In the current study, we have restricted our scientometric analysis to the most commonly used assays such as TUNEL, SCSA, Comet and SCD to understand the trends in SDF publication. Our results show that over the past 20 years, the TUNEL assay (*n* = 330) was most widely used to assess SDF, followed by SCSA (*n* = 265), Comet (*n* = 203) and SCD (*n* = 160) (Fig. 6). According to our results, TUNEL and SCSA assays were widely used in countries such as USA (*n* = 50 and *n* = 64, respectively) and Italy (*n* = 45 and n = 33, respectively), whereas Comet assay and SCD were employed mostly in the United Kingdom (n = 49) and Spain (*n* = 39), respectively (Table 2).

**Table 2:** Top institutions, countries, document type and journals based on SDF techniques

	TUNEL (*n* = 330)	SCSA (*n* = 265)	COMET (*n* = 203)	SCD (*n* = 160)
Institutions	Universita’ degli Studi di Firenze (*n* = 15)	Malmö University Hospital (*n* = 28)	Queen’s University Belfast (*n* = 26)	Universidad Autónoma de Madrid (*n* = 26)
	Royan Institute (*n* = 14)	ENEA Centro Ricerche Casaccia & South Dakota State University (*n* = 27)	University of Bradford (*n* = 17)	Royan Institute (*n* = 13)
	Inserm (*n* = 12)	Veterinary Research Institute, Brno (*n* = 14)	Institute of Clinical Science (*n* = 14)	Complejo Hospitalario Universitario Juan Canalejo (*n* = 11)
Countries	United States (*n* = 50)	United States (*n* = 64)	United Kingdom (*n* = 49)	Spain (*n* = 39)
	Italy (*n* = 45)	Italy (*n* = 33)	United States (*n* = 44)	China (*n* = 29)
	France (*n* = 44)	Sweden (*n* = 30)	China (*n* = 21)	Iran (*n* = 21)
Document Type	Original articles (*n* = 299)	Original articles (*n* = 228)	Original articles (*n* = 171)	Original articles (*n* = 151)
	Review (*n* = 16)	Review (*n* = 19)	Review (*n* = 19)	Review (*n* = 4)
	Others (*n* = 15)	Others (n = 18)	Others (*n* = 13)	Others (*n* = 5)
Journals	Human Reproduction (*n* = 41)	Fertility and Sterility (*n* = 32)	Human Reproduction (*n* = 26)	Fertility and Sterility (*n* = 23)
	Fertility and Sterility (*n* = 38)	Human Reproduction (*n* = 31)	Fertility and Sterility (*n* = 19)	Zhonghua Nan Ke Xue National Journal Of Andrology (*n* = 12)
	Andrologia (*n* = 24)	Andrology (*n* = 15)	Mutagenesis (*n* = 8)	Andrologia (*n* = 10)

### Step 6 - publication trends in SDF-based ART studies

**Fig. 7:**
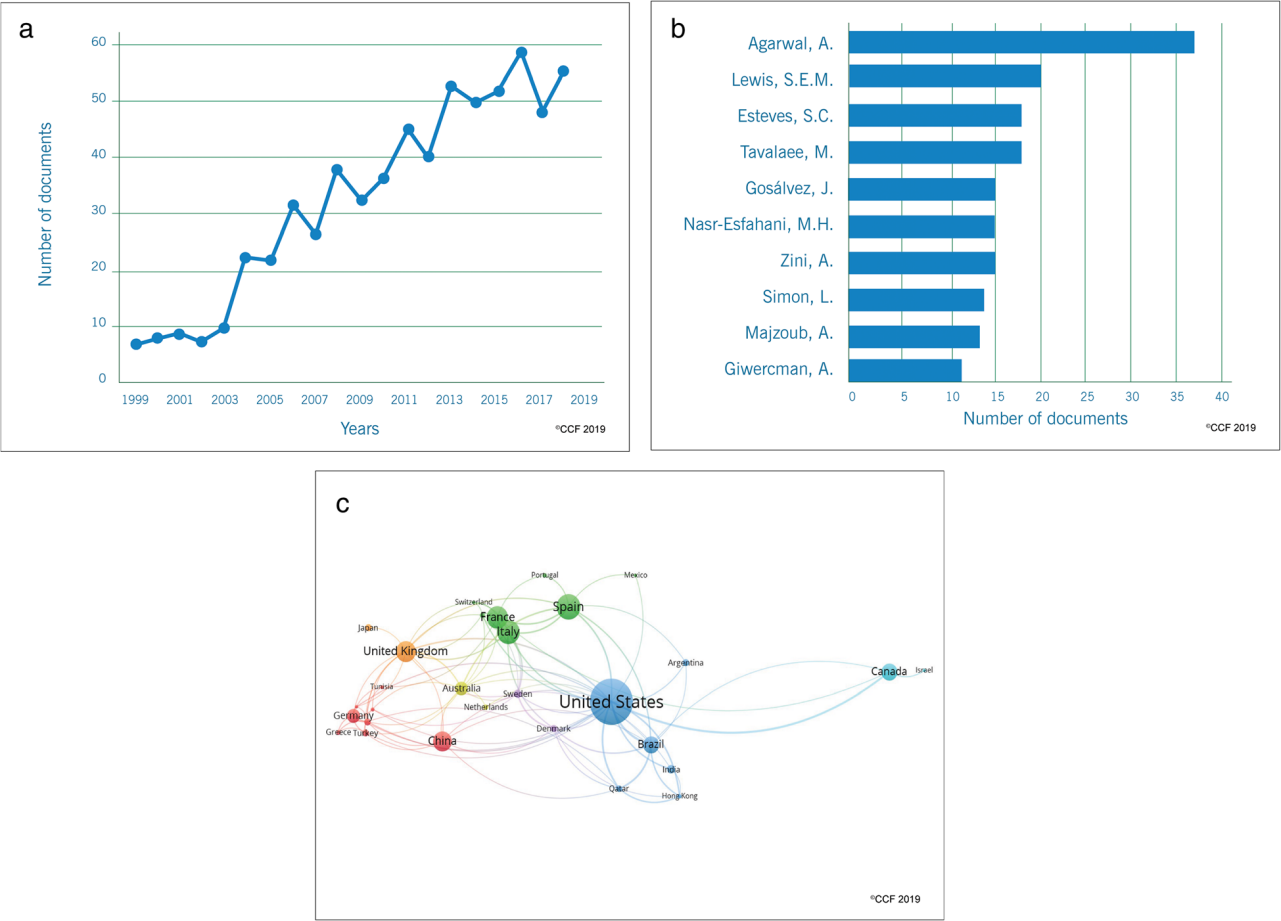
**a** Publication trend in SDF-based ART studies between1999–2018. **b** Top scientist involved in SDF-based ART studies. **c** SDF-based ART studies: Collaboration network

The linear increasing trend (R^2^ = 0.933) in the publications on SDF and ART over the past 20 years is represented in Fig. 7a. The articles were published in 154 different journals with the top 3 journals being Fertility and Sterility (*n* = 72), Human Reproduction (*n* = 62) and Reproductive Biomedicine Online (*n* = 54) accounting for 28.79% of the publications. The documents were mainly original articles (*n* = 480) and reviews (*n* = 116). All 159 authors had more than two publications with the top 3 contributors being Agarwal A., Lewis S.E.M. and Esteves S.C. (Fig. 7b). Cleveland Clinic Foundation (*n* = 36) in the USA, Royan Institute (*n* = 23) in Iran and Queen’s University Belfast (n = 23) in Northern Ireland were the top 3 institutions engaged in studies on SDF and ART. The USA was the most connected and productive country in terms of publications as illustrated in the network map (Fig. 7c). The countries which dominated the publications in the area of ART were the same as those for the prognostic/diagnostic studies, namely USA (*n* = 127), Spain (n = 62) and Italy (*n* = 58).

## Discussion

SDF is associated with male infertility and it adversely affects reproductive outcomes in couples [11, 31]. Both chromatin integrity and protamination status determine the extent of DNA damage [32] and tests have been used in the clinical laboratory settings to assess the SDF levels [33]. Though several studies have been published on SDF, scientometric analyses reported so far have not shed light on research trends in SDF. To our knowledge, this is the first methodical evaluation of SDF research based on scientometric approach. A stepwise analysis of the literature was conducted using the scientometric database from Scopus. The analysis of the literature revealed the extent of publications on SDF with respect to evaluation of male infertility, its importance as a diagnostic and prognostic tool in clinical scenarios, and in the field of ART (Table 3). Our results clearly illustrated the hot spots in research worldwide, the progress in this field as well as the applications of this parameter in the andrological practice.

**Table 3:** Key findings of the stepwise scientometric analysis of publications on SDF

Steps	Key findings
Step 1	Increased trend in publications on SDF over the past 20 years
Step 2	Increased number of articles reporting the association between SDF and male infertility
Step 3	SDF research was mainly focused on lifestyle, varicocele and asthenozoospermia while less investigated in association with testicular cancer, globozoospermia and hypogonadism
Step 4	Majority of the mechanistic studies were based on oxidative stress and the number of publications on mechanistic studies of SDF was twice as much as prognostic/diagnostic studies
Step 5	TUNEL assay was the most widely used technique to assess SDF
Step 6	Significant increase in publication trends of SDF-based ART studies

The integrity of DNA in sperm contributes significantly to fertilization and successful embryo development. In this context, SDF is evaluated as a measure of sperm DNA quality. In the current scientometric study, we observed an increasing trend in SDF publications in the past 20 years. The earlier techniques to assess SDF involved laborious and time-consuming manual counting of sperm under microscope, limiting its use in research [34]. With the advent of flow cytometry, it became possible to analyze more samples in less time with better accuracy, which may have contributed to the increase in publications [35]. Agarwal, A. was identified as the most productive author in terms of publications on SDF, and this was in agreement with the previous two bibliometric studies on male infertility [27, 36]. Fertility and Sterility (IF = 5.411) and Human Reproduction (IF = 5.506) are the top journals publishing studies on SDF (Fig. 2d). A large number of publications in these top cited journals indicate SDF as a hot topic being investigated extensively in the field of Andrology.

The decline in semen quality over the past few decades might explain the increased trend in male infertility research [1, 2, 37], as reported in our previous bibliometric study [27]. Semen analysis has been the routine test for the evaluation of male fertility potential since the first half of the twentieth century and has not overcome its limitations of being subjective and poor standardization [3, 38]. In addition, 10 to 30% of infertile patients are inexplicably normozoospermic [39]. Around 50% of infertile patients are classified as idiopathic, having abnormal semen parameters and normal physical and endocrine evaluation [40]. Therefore, the investigations have been moving towards identifying a new marker for sperm quality to support the routine semen analysis. In the current study, the increasing number of publications related to SDF and male infertility reveals the importance SDF has gained over the time in research and clinics as a test that adds value to evaluate male infertility.

Sperm DNA damage is being widely investigated in several clinical scenarios [27, 41]. In the current study, we observed that SDF evaluation was predominantly conducted in male infertility conditions associated with lifestyle, asthenozoospermia and varicocele. In this modern era, lifestyle choices are known to significantly influence male fertility potential [42]. The apparent decline in semen quality and an increase in incidence of testicular cancer in western men over the past century has been linked to lifestyle factors [43, 44]. According to the National Youth Tobacco Survey data published in 2018, the median age for smoking initiation in USA was reported to be 12.6 years [45], that coincides with the onset of puberty. Several studies have reported the adverse effects of smoking [46–49], alcohol abuse [42, 47, 49], and caffeine intake [49, 50] on semen quality and DNA integrity. In fact, the systematic review conducted by Ricci et al. suggested SDF as a possible mechanism by which caffeine intake causes male infertility [50]. These reports explain the increased number of publications linking lifestyle and SDF over the past 20 years. In addition, the adverse effect of lifestyle on male infertility could be overcome by better lifestyle choices, so this no-cost management could be one of the reasons for the increased interest in this field of research.

Asthenozoospermia, a common cause of male infertility, is characterized by reduced sperm motility [51] and has a prevalence of 18.71% [52]. Scientometric analysis revealed that the majority of SDF research was focused on asthenozoospermia compared to other sperm abnormalities such as asthenoteratozoospermia, oligoasthenozoospermia and oligoasthenoteratozoospermia. Several studies have reported a strong correlation between SDF and low motility [53, 54]. This explains the increasing number of publications on SDF that could facilitate the identification of its causative role in male infertility. Furthermore, our scientometric analysis revealed that varicocele was the third most investigated clinical scenario in SDF research. Varicocele is a pathology characterized by an enlargement of the pampiniform venous plexus and the internal spermatic veins, with an incidence of 15% in the healthy population and 40% in infertile men [55]. These patients show eight times higher levels of SDF compared to fertile donors [56, 57]. In fact, this pathology causes an increase in oxidative stress, which affects semen quality and reproductive outcomes [58, 59]. In addition, a significant improvement in SDF after varicocelectomy has been reported [12]. In this perspective, the evaluation of SDF could be of added value to routine semen analysis which explains the high number of SDF publications in this field.

On the contrary, lesser number of publications were observed on SDF with respect to testicular cancer, globozoospermia and hypogonadism. Testicular cancer particularly affects men of age 15 to 34 years and a total of 5.9 new cases/100000 men/year are reported by the National Cancer Institute (https://seer.cancer.gov/statfacts/html/testis.html), with an increasing incidence over the past few decades [60]. Although chemo- and radiotherapy have consequences on male fertility, currently they represent the gold standard therapy to treat cancer. Our previous bibliometric study identified testicular cancer as one of the top three research areas investigated in male infertility research [27]. Surprisingly, it is not so in case of SDF research despite the fact that chemo- and radiotherapy are well-known mutagenic agents with deleterious effects on sperm DNA integrity [61]. One possible explanation is that the patients are probably more focused on treating the condition than investigating their fertility status. Furthermore, a lot of progress has occurred in sperm cryopreservation techniques, which could bypass the issues due to chemotherapy-induced DNA damage that could probably explain the lower number of publications on SDF [62]. Globozoospermia is a rare condition with an incidence of less than 0.1% in the general population [63]. This small number of cases explain the lesser investigation on globozoospermia. Hypogonadism is a condition characterized by reduced synthesis of testosterone [64]. Clinical management of hypogonadism with hormone replacement therapy, treatments with clomiphene citrate and human chorionic gonadotropin are available [65], which could have undermined the necessity for additional research on sperm DNA integrity in hypogonadism.

Mechanistic and prognostic/diagnostic studies are considered as important step in bibliometric analysis [27]. In the current study, the number of documents on mechanistic studies (40.64%) were twice as much as prognostic/diagnostic studies (20.70%). Our results indicate that SDF was particularly investigated in correlation with oxidative stress-mediated male infertility since oxidative stress has been identified as one of the major causes of SDF [66, 67]. On the other hand, the lesser number of publications on apoptosis emphasize the need for more research to determine its role in SDF and effect on sperm function. The comparatively lesser publications on prognostic/diagnostic studies may be due to the increased focus on various other etiologies of male infertility. However, more research in the diagnostic/prognostic value of SDF is required to understand the clinical implications and management options.

There are several assays and laboratory tests available to assess the SDF [33]. The most commonly used tests, such as TUNEL, SCSA, Comet and SCD have been included in our scientometric analysis [33]. We found that TUNEL assay was the most widely used technique to assess SDF. Reportedly, the TUNEL assay is a sensitive and reliable method with minimal inter- and intra-observer variability [68, 69]. Hassanen et al. demonstrated an overall accuracy of 95.7% for TUNEL assay in determining SDF in infertile men [70]. In addition, several publications on standardization of the TUNEL assay in a clinical setup had raised the popularity of this test [68, 69].

Globally, the number of individuals conceived by ART has accelerated with time accounting for about 0.1% of the total population [71]. It is estimated that ART will be responsible for about 167 million lives i.e. 1.4% of the world population by 2100 [71]. Several studies have demonstrated the association of SDF with various ART reproductive outcomes, including embryo quality, fertilization, blastocyst formation rate, implantation and pregnancy rate [31, 72–74]. Furthermore, a few meta-analyses have emphasized the association between SDF and miscarriage rate, RPL and ART failure [31, 75, 76]. However, there are no clear-cut established predictive values for the SDF test. Therefore, there is a need for large prospective studies to assess the predictive value of SDF on reproductive outcomes in couples with male infertility factor. Our analysis disclosed a linear increase in the number of articles published, suggesting an increasing use of ART in couples with SDF. However, the relative distribution of publications on SDF was less in ART (30.78%) when compared to mechanistic studies (40.64%). This highlights the need for more ART-oriented research to facilitate the introduction of SDF into clinical practice as ART is the only management option currently available to overcome infertility in men having high SDF.

## Conclusions

Our analysis revealed an increasing trend in SDF publications over the past 20 years. SDF research trends were primarily focused on lifestyle, asthenozoospermia and varicocele while the less investigated areas were testicular cancer, globozoospermia and hypogonadism. Among the common methods used, the TUNEL assay was the most widely used technique to assess SDF. Currently, a substantial increase in research is essential to establish SDF as a prognostic/diagnostic parameter in the evaluation of clinical scenarios and ART outcomes.

## Supplementary information

**Additional file 1: Figure S1.** Number of publications in various clinical scenarios/risk factors associated with SDF in the past 20 years.

**Additional file 2: Figure S2.** Number of SDF publications associated with semen abnormality conditions.

**Additional file 3: Table S1.** Keywords used for each step in the Stepwise Model.

## Data Availability

All data generated or analyzed during this study are available from the corresponding author on reasonable request.
