# Supplementary figures and images for: Tracking research trends and hotspots in sperm DNA fragmentation testing for the evaluation of male infertility: a scientometric analysis
Source: Reprod Biol Endocrinol. 2019 Dec 26;17:110. doi: 10.1186/s12958-019-0550-3 (PMC6931248; doi:10.1186/s12958-019-0550-3)

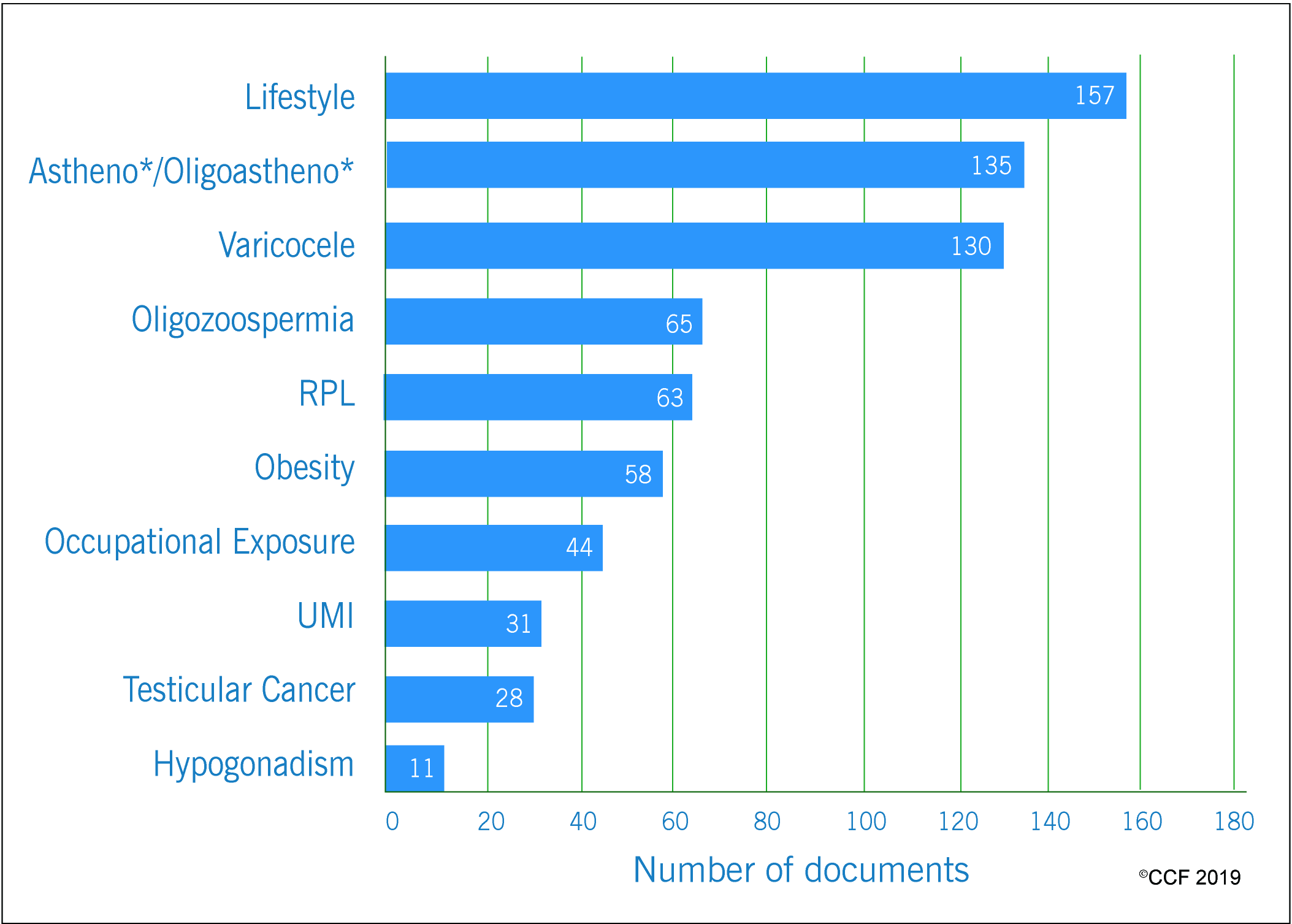

Supplement: Supplementary file 1 — Additional file 1: Figure S1. Number of publications in various clinical scenarios/risk factors associated with SDF in the past 20 years. [file 12958_2019_550_MOESM1_ESM.tif]

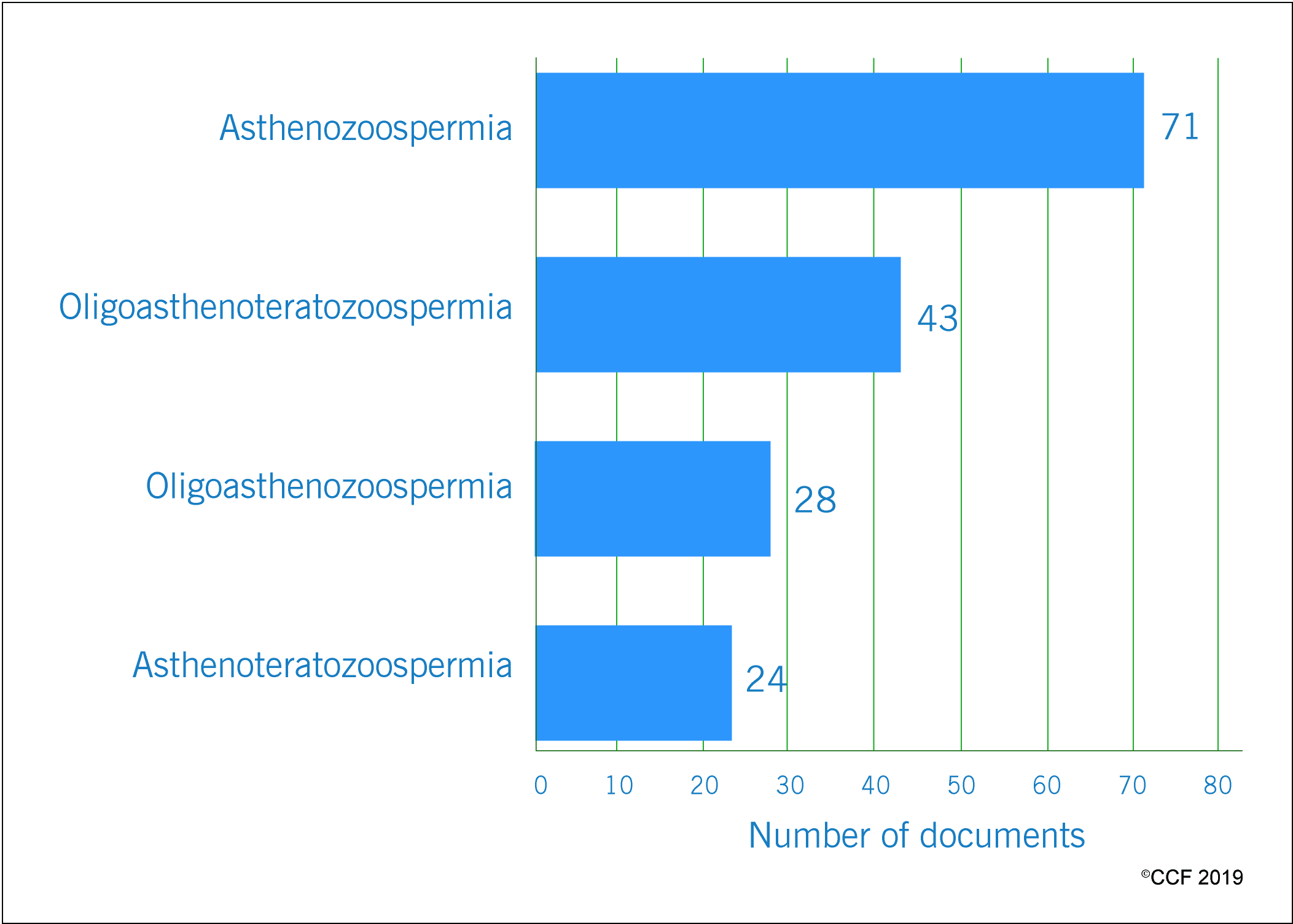

Supplement: Supplementary file 2 — Additional file 2: Figure S2. Number of SDF publications associated with semen abnormality conditions. [file 12958_2019_550_MOESM2_ESM.tif]
