# Supplementary material for: Tracking research trends and hotspots in sperm DNA fragmentation testing for the evaluation of male infertility: a scientometric analysis
Source: Reprod Biol Endocrinol. 2019 Dec 26;17:110. doi: 10.1186/s12958-019-0550-3 (PMC6931248; doi:10.1186/s12958-019-0550-3)
Supplement: Supplementary file 3 — Additional file 3: Table S1. Keywords used for each step in the Stepwise Model. [file 12958_2019_550_MOESM3_ESM.docx]

**Suppl. Table 1:** Keywords used for each step in the Stepwise Model

| **Steps** | **Content (1999-2018)** | **Keywords** |
| --- | --- | --- |
| Step 1 | SDF | (A) |
| Step 2 | SDF and Male Infertility | (A) AND TITLE-ABS ("infertil*" OR "subfertil*" OR "sterility") |
| Step 3 | Clinical Scenarios/Risk Factors |  |
|  | Varicocele | (A) AND TITLE-ABS ("varico*") |
|  | Oligozoospermia | (A) AND TITLE-ABS ("oligozoosperm*") |
|  | Astheno*/Oligoastheno* | (A) AND TITLE-ABS ("astheno*" OR "oligoastheno*") |
|  | Globozoospermia | (A) AND TITLE-ABS ("globozoo*") |
|  | Testicular Cancer | (A) AND TITLE-ABS ("testicular cancer" OR "testicular carcinoma" OR "testicular neoplasm" OR "testicular malignanc*" OR "testicular germ cell tumor") |
|  | UMI | (A) AND TITLE-ABS ("Unexplained male infertility" OR "Unexplained infertil*" OR "UMI") |
|  | Recurrent Pregnancy Loss | (A) AND TITLE-ABS ("recurrent" AND "pregnancy loss" OR "abortion" OR "spontaneous abortion" OR "miscarriage") |
|  | Hypogonadism | (A) AND TITLE-ABS ("hypogonad*") |
|  | Obesity | (A) AND TITLE-ABS ("obes*" OR "overweight" OR "BMI") |
|  | Lifestyle | (A) AND TITLE-ABS ("lifestyle" OR "smoking" OR "alcohol" OR "caffeine") |
|  | Occupational Exposure | (A) AND TITLE-ABS ("Occupational" OR "hazard" exposure) |
| Step 4 | Mechanistic Studies |  |
|  | Oxidative Stress | (A) AND TITLE-ABS ("oxidative stress" OR "reactive oxygen species" OR "ROS" OR "oxidative damage") |
|  | Apoptosis | (A) AND TITLE-ABS ("apoptosis" OR "caspase") |
|  | Sperm Function | (A) AND TITLE-ABS ("sperm function" OR "acrosome reaction" OR "capacitation" OR "sperm-oocyte fusion" OR "zona-pellucida binding" OR "zona binding") |
|  | Prognostic/Diagnostic Studies | (A) AND TITLE-ABS ("prognos*" OR "diagnos*" OR "predict*") |
| Step 5 | Evaluation Techniques |  |
|  | COMET | (A) AND TITLE-ABS ("COMET" OR "Single-cell gel electrophoresis" OR "SCGE") |
|  | SCSA | (A) AND TITLE-ABS ("SCSA" OR "Sperm chromatin structure assay") |
|  | SCD | (A) AND TITLE-ABS ("SCD" OR "Sperm chromatin dispersion" OR "Halosperm") |
|  | TUNEL | (A) AND TITLE-ABS ("TUNEL" OR " terminal deoxynucleotidyl transferase-mediated dUDP nick-end labelling") |
| Step 6 | ART | (A) AND TITLE-ABS ("assisted reproducti*" OR "ART" OR "IVF" OR "in vitro fertilization" OR "ICSI" OR "intracytoplasmic sperm injection") |

1. = (TITLE-ABS-KEY ( "sperm* DNA fragmentation" OR "Sperm* DNA damage" OR "Sperm* DNA integrity" OR "Sperm* chromatin damage" OR "Sperm* chromatin integrity" OR "sperm chromatin structure" OR "DNA fragmentation index" ) OR TITLE-ABS ( "sperm" OR "spermatozoa" AND "DNA strand break" OR "deoxyribonucleic acid fragmentation" OR "nuclear DNA damage" OR "DNA fragmentation" OR "DNA damage" ) AND TITLE-ABS-KEY ( "human" )
